# Diagnosis and management of long-bone nonunions: a nationwide survey

**DOI:** 10.1007/s00068-018-0905-z

**Published:** 2018-01-15

**Authors:** Sezai Özkan, Peter A. Nolte, Michel P. J. van den Bekerom, Frank W. Bloemers

**Affiliations:** 1 Department of Trauma Surgery, VU University Medical Center, VU University, de Boelelaan 1117, 1081 HV Amsterdam, The Netherlands; 2 Department of Orthopaedic Surgery, Spaarne Hospital, Hoofddorp, The Netherlands; 3 Department of Orthopaedic Surgery, Onze Lieve Vrouwe Kliniek, Amsterdam, The Netherlands

**Keywords:** Non-union, Delayed union, Fracture healing, Fracture

## Abstract

**Purpose:**

There is variability among surgeons on definitions regarding the degree of bone healing of long-bone fractures. A lack of consensus may negatively affect communication between surgeons, and lead to unintended and unwanted variability in treatment of patients suffering from abnormal healing of long-bone fractures. We aimed to identify differences between surgeons regarding their views on the degree of union of long-bone fractures.

**Methods:**

We performed a survey among 114 surgeons who worked at 11 level I trauma centers and 68 level II/III hospitals in the Netherlands. We asked them to represent their institutional colleagues and answer questions regarding their views on the definition, factors influencing bone healing, clinical practice, views on scientific evidence, and the use or need of guidelines for non-union of long-bone fractures. A total of 26 trauma surgeons and 37 orthopedic surgeons responded (59%).

**Results:**

Compared to trauma surgeons, more orthopedic surgeons maintain 6 months as the timeframe for classifying a fracture without healing tendencies as a non-union fracture (50 vs 70%; *P* = 0.019). Compared to orthopedic surgeons, trauma surgeons use the bone scan (46 vs 19%; *P* = 0.027) and the PET scan (50 vs 5.4%; *P* < 0.001) more often, and consider medication use to be a factor influencing bone healing more often (92 vs 69%; *P* = 0.040). Furthermore, they utilize bone marrow aspiration (35 vs 11%; *P* = 0.029), reaming of long bones (96 vs 70%; *P* = 0.010), synthetic bone substitutes (31 vs 5.4%; *P* = 0.012), bone morphogenetic proteins (58 vs 16%; *P* = 0.001), and the Diamond concept (92 vs 8.1%) more often as treatment modalities for non-union of long-bone fractures. Surgeons agreed on that intramedullary nail osteosynthesis was the treatment option supported by the highest level of evidence. 80% of the respondents feel a need for a clinical guideline on the management of long-bone non-union.

**Conclusion:**

There is no consensus among surgeons on the definition, factors influencing healing, clinical practice, and scientific evidence regarding non-union of long-bone fractures. The vast majority of surgeons believe that their practice would benefit from (inter)national guidelines on this topic, and efforts should be made to reduce surgeon-to-surgeon variability in treatment recommendations and facilitate more homogenous scientific research on non-union of long-bone fractures.

**Level of evidence:**

Level V.

## Introduction

Up to one in ten fractures of long bones may not show signs of bone union [1, 2]. Existing research identified factors that may influence bone healing after fractures [3]. These factors may be patient related (e.g. age, substance abuse status, nutritional status, comorbidities) [4–6], injury related (e.g. fracture severity, soft tissue damage, fracture site of the bone) [5, 7], or treatment related (e.g. operative vs non-operative management, plate fixation vs intramedullary nail fixation) [8, 9]. We may have an increasing understanding in factors influencing delayed union and non-union [10], but there is still no consensus on when a fracture is to be considered a delayed union or a non-union. In practice, this means that scientists, clinicians, and policy makers are referring to different entities when discussing delayed union or non-union of non-union fractures. The need of clear and uniform definitions for different degrees of fracture union was first emphasized almost 2 decades ago [11], and studies have highlighted the importance of a uniform definition of bone healing among surgeons thereafter [12, 13]. Unfortunately, up until today there seems to be no uniformity between surgeons regarding nomenclature in bone healing, which is in line with our experiences in clinical practice. In the Netherlands, both trauma surgeons and orthopedic surgeons treat non-union of long-bone fractures. We noticed that—among the different institutions of the authors—there still is no homogeneity on nomenclature regarding the degree of union of long bones among surgeons treating long-bone fractures. This negatively affects communication between physicians, scientists, and patients [11]. Therefore, through this study, we aimed to identify differences among Dutch surgeons’ views on degree of union of non-union fractures, more specifically on: (1) the definition, (2) factors influencing bone healing, (3) clinical practice (e.g. diagnostic work-up or treatment strategy), (3) views on scientific evidence regarding the treatment, and (4) the use and/or need of guidelines.

Our primary null-hypothesis is that there is no difference between trauma and orthopedic surgeons on the definition of non-union of long-bone fractures. Our secondary null-hypothesis is that there are no differences between trauma and orthopedic surgeons on factors influencing bone healing or work-up and management of long-bone fractures. Lastly, we hypothesized that there is no difference between trauma and orthopedic surgeons on views regarding scientific evidence on treatment modalities of long-bone non-unions or their needs for guidelines regarding the treatment of long-bone non-unions.

## Materials and methods

### Study design

We approached eligible surgeons for participation in this cross-sectional nationwide study in July 2016. Based on our national regulations, this study did not require Institutional Review Board approval for this survey study where the obtained information was recorded in a de-identified manner. Our survey questionnaire (Appendix A) was distributed among 114 surgeons who worked at 11 level I trauma centers and 68 level II/III hospitals in the Netherlands. The surgeons were specifically asked to represent their institutional colleagues treating non-union of long-bone fractures by choosing answers that best reflect the general agreement among their colleagues. The participants were approached by e-mail, and those without a (timely) response were sent reminders after two and four months, respectively.

### Questionnaire

The questionnaire referred to various aspects of the treatment of symptomatic non-unions of long-bone fractures. The questionnaire was designed by a team comprising two orthopedic surgeons and 1 trauma surgeon, who are all experienced in treating non-unions and have experience in scientific research on this topic. An electronic survey platform was used to distribute the survey and to receive responses anonymously. This questionnaire was sent along with an instruction page.

Participants provided basic socio-demographic information and were assessed for their clinical views on non-union. The socio-demographic information consisted of age, subspecialty, institution type, and annual treatment volume. The study-specific questionnaire consisted of 12 questions concerning the work-up and treatment of long-bone non-unions and the scientific evidence and guidelines on this topic. More specifically, the survey was based on the definition, diagnosis, prognostic factors, and treatment of non-unions (Appendix A). The options for the question regarding the scientific foundations for choosing a treatment modality were posed in a seven-point Likert-scale, ranging from seven points for meta-analysis to one point for expert opinion.

### Statistical analysis

Categorical data were presented as frequencies with percentages and continuous data as means with standard deviations. The differences in characteristics of the participating surgeons (Table 1) were analyzed using the Fisher’s exact test. The differences between surgeon responses to dichotomous or categorical and continuous variables were assessed with the Fisher’s exact test and the two-sample student *t* test with equal variance, respectively. A *P* value of < 0.05 was considered statically significant.

**Table 1:** Characteristics of participating surgeons (*n* = 63)

	All surgeons	Surgical subspecialty		*P* value
	(*n* = 63)	Trauma (*n* = 26)	Orthopaedic (*n* = 37)	
Age category				0.70^1^
30–40 years	13 (21)	7 (27)	6 (16)	
40–50 years	22 (35)	9 (35)	13 (35)	
50–60 years	21 (33)	7 (27)	14 (38)	
> 60 years	7 (11)	3 (12)	4 (11)	
Institution type				0.48^1^
General hospital	29 (46)	10 (38)	19 (51)	
Categorical hospital	1 (1.6)	0	1 (2.7)	
Tertiary hospital	22 (35)	10 (38)	12 (32)	
University Medical Center	10 (16)	6 (23)	4 (11)	
Private practice	1 (1.6)	0	1 (2.7)	
Treatment volume				< 0.001^**1**^
< 5 patients	27 (43)	1 (3.9)	26 (70)	
5–10 patients	18 (29)	9 (35)	9 (24)	
10–15 patients	9 (14)	7 (27)	2 (5.4)	
15–20 patients	6 (9.5)	6 (23)	0	
> 20 patients	3 (4.8)	3 (12)	0	

^1^Fisher’s exact test

### Participant characteristics

67 surgeons (59%) responded to our questionnaire. After excluding four surgeons due to not answering the study-specific questionnaire, our final cohort consisted of 26 trauma surgeons and 37 orthopedic surgeons. All participating surgeons in our study treated patients with long-bone non-unions. Most participating surgeons (35%) were between 40 and 50 years old and worked in a general hospital (35%). The two groups were comparable in terms of age and institution type, but the participating trauma surgeons had a higher volume regarding the treatment of non-union of long-bone fractures (Table 1).

## Results

Compared to the group of trauma surgeons, more orthopedic surgeons in this study maintain 6 months as the timeframe for classifying a painful fracture without healing tendencies as a non-union fracture (70 vs 50%; *P* = 0.019; Table 2). Six trauma surgeons (23%) chose ‘other than above’ when asked for the timeframe used for defining non-union of long-bone fractures. Of these, two surgeons stated that their definition depends on the specific bone, one surgeon stated that it depends on the mechanism of injury, one surgeon maintains four months as the timeframe, one surgeon mentioned ‘physiological timeframe’, and one surgeon stated ‘between 4 and 6 months’.

**Table 2:** Definition of pseudoarthrosis, diagnostic modalities used, and factors influencing bone healing (*n* = 63)

	All surgeons	Surgical subspecialty		*P* value
	(*n* = 63)	Trauma (*n* = 26)	Orthopaedic (*n* = 37)	
Definition of pseudoarthrosis				0.019^***1***^
3 months	6 (9.5)	2 (7.7)	4 (11)	
6 months	39 (62)	13 (50)	26 (70)	
9 months	12 (19)	5 (19)	7 (19)	
Other than above	6 (9.5)	6 (23)	0	
Diagnostic modalities used				
Radiograph	57 (90)	25 (96)	32 (86)	0.39^1^
Echo	1 (1.6)	0	1 (2.7)	> 0.99^1^
CT-scan	61 (97)	26 (100)	35 (95)	0.51^1^
MRI	12 (19)	8 (31)	4 (11)	0.058^1^
Bonescan	19 (30)	12 (46)	7 (19)	0.027^***1***^
PET	15 (24)	13 (50)	2 (5.4)	< 0.001^***1***^
Rule out infection	40 (63)	19 (73)	21 (57)	0.29^1^
Serology: biomarkers	2 (3.2)	1 (3.9)	1 (2.7)	> 0.99^1^
Other than above	3 (4.8)	1 (3.9)	2 (5.4)	> 0.99^1^
Patient characteristics influencing bone healing				
Age	33 (52)	16 (62)	17 (46)	0.31^1^
ASA-classification	24 (38)	12 (46)	12 (32)	0.30^1^
Malnutrition	58 (92)	26 (100)	32 (86)	0.071^1^
Diabetes Mellitus	37 (59)	18 (69)	19 (51)	0.20^1^
Anaemia	13 (21)	8 (31)	5 (14)	0.12^1^
Growth-hormone deficiency	11 (17)	7 (27)	4 (11)	0.18^1^
Alcohol abuse	39 (62)	17 (65)	22 (59)	0.79^1^
Tobacco abuse	62 (98)	25 (96)	37 (100)	0.41^1^
Medication (e.g. Antibiotics, corticosteroids)	24 (38)	15 (58)	9 (24)	0.009^***1***^
Pathological bone	52 (83)	18 (69)	34 (92)	0.040^***1***^
Other than above	8 (13)	6 (23)	2 (5.4)	0.056^1^
Fracture characteristics influencing bone healing				
Soft tissue injury	61 (97)	26 (100)	35 (95)	0.51^1^
Open fracture	58 (92)	24 (92)	34 (92)	> 0.99^1^
Location (epiphysis, metaphysis, diaphysis)	56 (89)	23 (88)	33 (89)	> 0.99^1^
Amount of displacement	51 (81)	23 (88)	28 (76)	0.33^1^
Compartment syndrome	23 (37)	9 (35)	14 (38)	> 0.99^1^
Other than above	6 (9.5)	2 (7.7)	4 (11)	> 0.99^1^

^1^Fisher’s exact test

When asked for the diagnostic modalities used, trauma surgeons reported to use the bone scan (46 vs 19%; *P* = 0.027) and the PET scan (50 vs 5.4%; *P* < 0.001) more often than orthopedic surgeons (Table 2). Of the three surgeons that also chose ‘other than above’ as diagnostic modalities, two orthopedic surgeons mentioned clinical signs and one trauma surgeon mentioned lab-results of the osteoporosis screening.

With regards to patient characteristics, trauma surgeons consider medication use to be a factor that influences bone healing more often than orthopedic surgeons (58 vs 24%; *P* = 0.009), whereas orthopedic surgeons consider pathological bone to influence bone healing more often than trauma surgeons (92 vs 69%; *P* = 0.040; Table 2). Of the eight surgeons who also considered other patient characteristics to influence bone healing, one orthopedic surgeon mentioned patient behavior, and one surgeon mentioned infection. Of the remaining trauma surgeons, one surgeon mentioned vitamin D deficiency, three surgeons mentioned fracture instability, and two surgeons mentioned NSAID use.

There was no difference between the groups with regards to views on fracture characteristics that influence bone healing (Table 2). Of the six surgeons who also chose ‘other than above’, two trauma surgeons and one orthopedic surgeon mentioned fracture stability. The remaining three orthopedic surgeons mentioned insufficient bone contact, vascular status, and post-radiotherapy tissue as additional fracture characteristics that may influence bone healing.

Compared to orthopedic surgeons, trauma surgeons utilize bone marrow aspiration (35 vs 11%; *P* = 0.029), reaming of long bones (96 vs 70%; *P* = 0.010), synthetic bone substitutes (31 vs 5.4%; *P* = 0.012), bone morphogenetic proteins (58 vs 16%; *P* = 0.001), and the Diamond concept (92 vs 8.1%) more often as treatment modalities for non-union of long-bone fractures (Table 3). Two trauma surgeons mentioned that they also use frequency rhythmic modulation system and parathyroid hormone as a treatment modality. One orthopedic surgeon reported to also use the masquelet technique for the treatment of non-union of long-bone fractures.

**Table 3:** Treatment modalities used for treatment of long-bone non-union (*n* = 63)

Treatment modality, *n* (%)	All surgeons	Surgical subspecialty		*P* value
	(*n* = 63)	Trauma (*n* = 26)	Orthopaedic (*n* = 37)	
Autologeous bone	56 (89)	23 (88)	33 (89)	> 0.99^1^
Bonemarrow aspiration	13 (21)	9 (35)	4 (11)	0.029^***1***^
Reaming of long bones	51 (81)	25 (96)	26 (70)	0.010^***1***^
Synthetic bone substitutes	10 (16)	8 (31)	2 (5.4)	0.012^***1***^
Allograft	28 (44)	12 (46)	16 (43)	> 0.99^1^
Bone morphogenetics proteins	21 (33)	15 (58)	6 (16)	0.001^***1***^
Platelets Rich Plasma	2 (3.2)	1 (3.9)	1 (2.7)	> 0.99^1^
Low intensity ultrasound	20 (32)	9 (35)	11 (30)	0.79^1^
Pulsed Electromagnetic Field	14 (22)	7 (27)	7 (19)	0.54^1^
Shockwave therapy	1 (1.6)	0	1 (2.7)	> 0.99^1^
Operative: debridement + plate fixation	61 (97)	25 (96)	36 (97)	> 0.99^1^
Operative: IM-pin osteosynthesis	57 (90)	25 (96)	32 (86)	0.39^1^
Operative: dynamic IM-pin	58 (92)	25 (96)	33 (89)	0.39^1^
Operative: bone lengthening/shortening	23 (37)	13 (50)	10 (27)	0.071^1^
Diamond concept	27 (43)	24 (92)	3 (8.1)	< 0.001^***1***^
Plaster cast or brace	28 (44)	13 (50)	15 (41)	0.61^1^
Other	3 (4.8)	2 (7.7)	1 (2.7)	0.37^1^

^1^Fisher’s exact test

An overview of the responses to our question ‘What is your most important reason for not using the previously mentioned treatment modalities?’ is presented in Table 4.

**Table 4:** Motivation for not utilizing treatment modalities for the treatment of long-bone non-union (*n* = 63)

Treatment modality, *n* (%)	Insufficient funds		Insufficient evidence		Personal beliefs		Unfamiliar with technique		Inexperienced		Other	
	Trauma	Ortho	Trauma	Ortho	Trauma	Ortho	Trauma	Ortho	Trauma	Ortho	Trauma	Ortho
Autologeous bone	0	0	1 (3.9)	1 (2.7)	–	0	0	0	0	0	2 (7.7)	2 (5.4)
Bonemarrow aspiration	0	0	4 (15)	9 (24)	2 (7.7)	4 (11)	1 (3.9)	8 (22)	9 (35)	10 (27)	1 (3.9)	1 (2.7)
Reaming of long bones	0	2 (5.4)	0	3 (8.1)	0	0	0	1 (2.7)	0	4 (11)	1 (3.9)	1 (2.7)
Synthetic bone substitutes	1 (3.9)	1 (2.7)	4 (15)	10 (27)	4 (15)	7 (19)	2 (7.7)	6 (16)	6 (23)	7 (19)	1 (3.9)	3 (8.1)
Allograft	0	3 (8.1)	2 (7.7)	2 (5.4)	4 (15)	6 (16)	1 (3.9)	1 (2.7)	4 (15)	2 (5.4)	3 (12)	6 (16)
Bone morphogenetics proteins	6 (23)	12 (32)	2 (7.7)	8 (22)	2 (7.7)	1 (2.7)	0	6 (16)	1 (3.9)	3 (8.1)	0	0
Platelets Rich Plasma	1 (3.9)	2 (5.4)	12 (46)	17 (46)	1 (3.9)	9 (24)	1 (3.9)	4 (11)	10 (39)	3 (8.1)	0	0
Low intensity ultrasound	0	0	8 (31)	11 (30)	3 (12)	3 (8.1)	0	5 (14)	4 (15)	5 (14)	2 (7.7)	1 (2.7)
Pulsed Electromagnetic Field	0	1 (2.7)	12 (46)	11 (30)	2 (7.7)	5 (14)	2 (7.7)	6 (16)	2 (7.7)	5 (14)	1 (3.9)	2 (5.4)
Shockwave therapy	0	2 (5.4)	12 (46)	14 (38)	4 (15)	12 (32)	2 (7.7)	1 (2.7)	7 (27)	5 (14)	1 (3.9)	1 (2.7)
Operative: debridement + plate fixation	0	0	0	0	0	0	0	0	0	0	1 (3.9)	1 (2.7)
Operative: IM-pin osteosynthesis	0	0	0	0	0	1 (2.7)	0	0	0	0	1 (3.9)	4 (11)
Operative: dynamic IM-pin	0	0	0	1 (2.7)	1 (3.9)	0	0	0	0	1 (2.7)	0	2 (5.4)
Operative: bone lengthening/shortening	0	0	0	0	0	0	2 (7.7)	4 (11)	6 (23)	12 (32)	5 (19)	10 (27)
Diamond concept	1 (3.9)	2 (5.4)	0	2 (5.4)	0	0	1 (3.9)	23 (62)	0	3 (8.1)	0	3 (8.1)
Plaster cast or brace	0	0	1 (3.9)	3 (8.1)	7 (27)	5 (14)	0	0	0	2 (5.4)	5 (19)	11 (30)

There was no difference between the groups in views on scientific evidence regarding the different treatment modalities. Both groups believed that intramedullary nail osteosynthesis was the treatment option supported by the highest level of evidence (Table 5).

**Table 5:** Evidence levels assigned to treatment modalities (*n* = 53)

Treatment modality, *n* (%)	All surgeons	Surgical subspecialty		*P* value
	(*n* = 53)	Trauma (*n* = 22)	Orthopaedic (*n* = 31)	
Autologeous bone	4.9 ± 1.5	5.0 ± 1.3	5.0 ± 1.6	0.93^1^
Bonemarrow aspiration	4.2 ± 1.3	4.4 ± 1.0	4.0 ± 1.5	0.36^1^
Reaming of long bones	5.0 ± 1.3	5.1 ± 1.1	5.0 ± 1.4	0.89^1^
Synthetic bone substitutes	4.3 ± 1.6	4.2 ± 1.5	4.4 ± 1.7	0.67^1^
Allograft	4.8 ± 1.5	4.3 ± 1.5	5.1 ± 1.4	0.078^1^
Bone morphogenetics proteins	5.2 ± 1.4	5.7 ± 0.98	4.9 ± 1.6	0.050^1^
Platelets Rich Plasma	3.8 ± 1.4	3.6 ± 1.4	4.0 ± 1.5	0.39^1^
Low intensity ultrasound	4.2 ± 1.9	3.8 ± 1.6	4.6 ± 2.0	0.17^1^
Pulsed Electromagnetic Field	4.2 ± 1.7	4.0 ± 1.7	4.4 ± 1.7	0.37^1^
Shockwave therapy	3.8 ± 1.7	3.7 ± 1.8	3.9 ± 1.7	0.77^1^
Operative: debridement + plate fixation	5.4 ± 1.4	5.6 ± 1.3	5.2 ± 1.5	0.44^1^
Operative: IM-pin osteosynthesis	5.6 ± 1.2	5.7 ± 1.0	5.5 ± 1.3	0.56^1^
Operative: dynamic IM-pin	5.1 ± 1.4	5.6 ± 1.1	4.9 ± 1.6	0.098^1^
Operative: bone lengthening/shortening	4.5 ± 1.3	4.6 ± 0.86	4.5 ± 1.5	0.92^1^
Diamond concept	4.8 ± 1.8	5.2 ± 1.8	4.4 ± 1.8	0.21^1^
Plaster cast or brace	4.7 ± 1.4	4.5 ± 1.3	4.8 ± 1.5	0.47^1^

^1^Student *t* test

Over half of the study participants does not use an algorithm for choosing a treatment modality in the treatment of non-union and 83% of the study participants feels a need for an algorithm or a national guideline for the treatment of non-union (Table 6). There was no difference between the two groups on this subject. When asked to comment on their choice, surgeons stated that their care would benefit from a more evidence based and uniform treatment. The main motivation of the participants who felt a need for an app/tool to estimate the risk of non-union was to incorporate this tool in their patient education.

**Table 6:** Surgeons’ views on guidelines and algorithms regarding the treatment of long-bone non-union (*n* = 53)

	All surgeons	Surgical subspecialty		*P* value
	(*n* = 53)	Trauma (*n* = 22)	Orthopaedic (*n* = 31)	
Uses algorithm for choice of treatment modality				0.11^1^
Yes, made available by institution	11 (21)	7 (32)	4 (13)	
Yes, developed individually	9 (17)	5 (23)	4 (13)	
No	33 (62)	10 (45)	23 (74)	
Feels need for algorithm for choise of treatment modality	44 (83)	16 (73)	28 (90)	0.14^1^
Feels need for app/tool to estimate risk on non-union	32 (60)	11 (50)	21 (68)	0.26^1^
Feels need for development of national guideline on treatment of non-union	44 (83)	18 (82)	26 (84)	> 0.99^1^

^1^Fisher’s exact test

## Discussion

There is a lack of consensus on definitions of union, delayed union, and non-union of long-bone fractures. This negatively affects communication between surgeons [11–14], and may lead to surgeon-to-surgeon variability in the management of long-bone fractures. Through this cross-sectional study, we aimed to identify differences between surgeons regarding their views on the degree of union of long-bone fractures.

This study must be interpreted in light of its strengths and limitations. We had a response rate of 59% and therefore, there may be some nonresponder bias. This is, however, inherent to this type of study. Medical specialists are known to show a high variability in response rates [15], and our response rate is in line with other comparable studies [12, 13]. Second, this study is conducted in the Netherlands and repeating this study in countries with different surgical training programs or different (utilization of) healthcare resources may lead to different outcomes. We expect this to have a minimal effect on the external validity of our study based on prior research [12]. Lastly, the trauma surgeons had a higher treatment volume of non-union of long-bone fractures than the orthopedic surgeons in this study. Prior research on surgeon notions of non-union showed that treatment volume did not affect the surgeons’ definition of non-union and we, therefore, do not expect this to bias our results [13].

Our results show that surgeons in the Netherlands maintain different timeframes for the definition of non-union (Table 2). This high variability in definitions of non-union of long-bone fractures is consistent with prior studies [12, 13], although those studies did not specifically report differences between surgical subspecialties.

Compared to orthopedic surgeons, trauma surgeons were more likely to use imaging modalities based on biological activity such as bone scintigraphy and positron emission tomography (PET) as a diagnostic imaging modality for non-union of long-bone fractures. Bone scintigraphy has been studies historically for detecting abnormal bone healing [16], and may have a role complementary to medical history taking, clinical assessment, and radiographic assessment of fractures with compromised union [17]. Animal studies have shown the potential of PET imaging as an indicator of fracture non-union [18], and PET might be useful in differentiating infected from non-infected non-unions when clinical findings for local infection are inconclusive [19].

There was a difference in views on patient characteristics that may influence bone healing between trauma surgeons and orthopedic surgeons. Trauma surgeons considered the use of medication as a prognostic factor for bone healing more often than orthopedic surgeons, whereas orthopedic surgeons considered pathological bone to be a prognostic factor more often (Table 2). The use of medication, corticosteroids specifically, has been identified as a moderate contributor to fracture non-union in another cross-sectional study among orthopedic surgeons [12].

Trauma surgeons and orthopedic surgeons agreed on fracture characteristics that may influence bone healing. An earlier study showed that, indeed, surgeons agreed on the prognostic abilities of fracture characteristics such as morphology and the degree of soft-tissue injury [12].

Our results indicate that both trauma surgeons and orthopedic surgeons utilize a wide array of treatment modalities (Table 3). Orthopedic surgeons utilize bone marrow aspiration, reaming of long bones, synthetic bone substitutes, bone morphogenetics proteins, and diamond concept less often than trauma surgeons. While prior studies assessing at surgeon agreement on definition, perceived causes, and assessment of long-bone non-union [12, 13], to our best knowledge no studies have assessed the variability in terms of treatment choice among surgeons. As the etiology of long-bone non-union is multifaceted, patients suffering from non-union require a tailored approach [20, 21].

When asked for their motivation for not utilizing treatment modalities in the clinical management of non-union of long-bone fractures, a lack of financial resources seemed to play a minor role, whereas insufficient evidence was the predominant reason for not utilizing treatment modalities (Table 4). This surgeons’ perceived lack of evidence for these treatment modalities may be a symptom of the high variability in the assessment of fracture-healing in orthopedic trauma studies: the interpretation of fracture care studies remains difficult due to different definitions regarding key concepts in fracture care [14].

The surgeons did not have different views on levels of evidence assigned to the treatment modalities (Table 5). Interpretation of studies regarding care of non-union of long-bones is, however, difficult as there is a high variability in the assessment of fracture-healing in orthopedic trauma studies, inevitably leading to bias [14].

Most of the surgeons in this study feels a need for an algorithm as an aid in clinical decision-making for the treatment of non-union of long-bone fractures (Table 6). Despite classification systems being proposed almost a decade ago [22], the need for standardization has been emphasized repeatedly [5, 12], and the results of this survey show that this need has yet to be fulfilled.

In conclusion, our results indicate that surgeons maintain different definitions for non-union of long-bone fractures. This may bias clinical studies, facilitate miscommunications between surgeons (and their patients), and contribute to surgeon-to-surgeon treatment variability. The lack of standardization in this matter is a long-lasting [11], international [12], and—as our results indicate—interdisciplinary problem. Non-union of long-bones is a complex concept. It is a continuous outcome, rather than a dichotomous yes-or-no result, and comprises both radiological and biological modalities. Due to its complexity, treatment of non-union fractures requires a multi-faceted strategy [20, 21]. The treatment options for non-union of non-union fractures evolve continuously [10], and the surgeons’ treatment recommendation is the result of a mixture of clinical evidence, experience, and personal beliefs. Our results underline the complexity of this subject and illustrate that—despite consensus on some aspects of long-bone non-union fracture care—there is heterogeneity among surgeons in the Netherlands with regards to this topic. To facilitate well-considered and evidence-based surgical care, standardization in definitions is, still, necessary. Efforts should be made to provide clinical tools such as (inter)national guidelines on management of long-bone non-unions. As surgeons would benefit from this, hopefully, this will reduce surgeon-to-surgeon variability in treatment recommendations, facilitate more homogenous scientific research, and lead to more standardized care of patients suffering from non-union of long-bone fractures.

## Appendix A

### Questionnaire Survey

1. Which diagnostic modalities do you use in the work-up of delayed or non-unions of long bones?
2. Which patient characteristics are important in the treatment of delayed or non-unions of long bones?
3. Which fracture characteristics are important in the development of delayed or non-unions of long bones?
4. Which treatment modalities do you use in the treatment of delayed or non-unions of long bones?
5. What is your main motivation for not using a specific treatment?
6. What is the level of evidence to support the several treatment options?
7. Do you use an algorithm to guide the treatment of delayed or non-unions of long bones?
8. Do you desire a scientifically based treatment algorithm?
9. Do you desire an app to predict the risk of non-union to inform the treating surgeon and the patient?
10. Do you desire a national guideline to guide the treatment of non-unions of long bones?
11. Do you want to be involved in developing this national guideline?
12. Do you desire education concerning the work-up and treatment of long bone non-unions?
